# Perceptions and Practice of Labor Pain-Relief Methods among Health Professionals Conducting Delivery in Minia Maternity Units in Egypt

**DOI:** 10.1155/2018/3060953

**Published:** 2018-09-26

**Authors:** Ola Mousa, Amal Ahmed Abdelhafez, Ahmed R. Abdelraheim, Ayman M. Yousef, Ahmed A. Ghaney, Saad El Gelany

**Affiliations:** ^1^Faculty of Nursing, Minia University, Minia, Egypt; ^2^Obstetrics and Gynecology Department, Faculty of Medicine, Minia University, Egypt

## Abstract

**Introduction:**

In low-resource settings (LRSs), pain relief during labor is often neglected. Women and health professionals (HPs) may lack awareness of analgesic options, may not accept these options, or may have concerns regarding their safety. Furthermore, even if women or HPs preferred labor analgesia, options may not be available at the hospital. This study was carried out to explore how HPs perceive and practice pain management during labor in Minia maternity units in Egypt.

**Methods:**

A structured, self-administered questionnaire from 306 HPs in Minia maternity units from August 1, 2016, to August 30, 2017, after approval by the organizational Ethical Review Committee.

**Results:**

The response rate was 76.5%. The majority, 78.2% of participants, believed in pain relief during labor. However, their practices are different. In the first stage of labor, almost 44.9% used nonpharmacological methods, whereas 36.8% used neither pharmacological nor nonpharmacological methods. Hospital-related factors were the major barriers against using pain-relief methods, as stated by HPs.

**Conclusion:**

Although most HPs understand the role of analgesia in labor pain relief, there is a wide gap between the use of pain-relief methods and women's need in Minia, Egypt; HPs claim this is due to health care facilities. There is an urgent need to identify the barriers against and raise the awareness among the community and HPs of the need to use pain-relief methods as part of improving the quality of care during labor.

## 1. Introduction

Cultural and social modifiers showed a great interest in pain relief during labor [1]. Although giving birth is the most extreme feeling a woman can feel physically, it can be a very painful experience due to many factors, such as uterine ischemia in the first stage and stretching of the vagina and perineum and compression of pelvic structures during the second stage [2].

Awareness of labor pain in a multidimensional framework puts an emphasis on a woman-centered method of labor pain management that involves an extensive scope of pharmacological and nonpharmacological approaches [3].

The criteria for the ideal method of pain relief during labor should be safe and effective and not affect either the women's mobility or the progress of labor and should be in a woman-centered environment [4].

In high-income countries (HICs), pain relief during labor is an integral part of the labor process [5], and there is a wide range of methods to relieve pain by pharmacological means, such as oral tablets, inhalation analgesia, intravenous and intramuscular opioids (pethidine or diamorphine), and various types of local (paracervical or pudendal block) and regional (epidural or spinal anesthetic) analgesia, and nonpharmacological ways, such as relatives' support, breathing exercises, massage, water birth, and the use of transcutaneous electrical nerve stimulation (TENS) in early labor.

The situation in low-resource settings (LRSs) is different; pain relief during labor is often neglected apart from relatives' support [6] because labor pain is considered a natural process and women should be able to cope. The reported reasons behind lack of provision of pain-relief methods, especially of pharmacological methods, in LRSs are lack of awareness from both health professionals (HPs) and women, nonacceptance, unavailability, and safety concerns [6, 7].

From a humane viewpoint and based on the subjectivity of pain relief, every woman should have her own choice of pain relief during labor [5].

One of the factors that affect delivery in public hospitals is the lack of physicians' support and inadequate quality that is related to pain relief [8]. Thus, awareness by HPs has a key role in supporting women's choice and access to pain-relief options during labor. Many factors could affect the woman's choice of labor pain relief, including her expectations, HPs' support, and her involvement in the decision-making [9].

Labor pain management is now accepted and implemented in many countries of the world; however, in Egypt, pain management during labor is not yet commonly practiced. Thus, this study was conducted to discern the use of obstetric analgesia in the management of pain during labor and delivery among obstetric caregivers in the Minia Maternity and Children University Hospital.

Our objective is to explore the different methods obstetricians and nurses, who conduct normal vaginal delivery, use to relieve pain, and their perception of those methods.

## 2. Materials and Methods

### 2.1. Study Settings

This is an institution-based, cross-sectional study: a paper-based, structured questionnaire survey, conducted in maternity units in Minia Governorate, including Minia Maternity and Children University Hospital (the only tertiary hospital in El Minia Governorate, serving a population of 5.5 million and providing comprehensive emergency obstetric care) and nine district maternity units from August 1, 2016, until August 30, 2017.

### 2.2. Study Population

Three hundred six obstetric HPs (OBGYN qualifications) were invited, and a written informed consent was obtained after clarification of the purpose of the study; the guidelines by which to complete the questionnaire, ensuring confidentiality of the responses; and the right to withdraw the results at any time for no reason. Out of the 306 HPs approached, 234 of them returned completed questionnaires.

### 2.3. Data Collection and Analysis Methods

A structured, self-administrated questionnaire was conducted individually. The purposive sample was made of participants who were full-time employees at the designated study location, including obstetricians who worked with mothers in labor. They were asked verbally for voluntary participation.

Contact details of the principal investigator and a research assistant were provided in case a participant had questions or concerns that arose during the study period.

### 2.4. Ethical Considerations

The research project was approved by the ethical committee of the Department of Obstetrics and Gynecology, Minia University Hospital, on April 10, 2015 (registration number: MU 7219). Written informed consent was collected from the participants after clarification of the purpose of the questionnaire; the guidelines by which to complete it, ensuring confidentiality of the responses; and the right to withdraw the results at any time for no reason.

Tools used in the study were developed by the researchers after review of the related current local and international literature, using books, articles, and scientific journals.

For data collection, a structured interview questionnaire was used to assess the following:

- The sociodemographic characteristics of the obstetricians, including age, gender, hospital, working experience, and training in pain-relief methods during a patient's labor
- Participants' perception and attitude toward labor pain-relief methods
- Participants' use of labor pain-relief methods
- Barriers to using labor pain-relief methods in health care settings, using the four-point Likert scale (agree, strongly agree, disagree, and strongly disagree)

A pilot study was conducted with 6 HP participants from the abovementioned settings to measure the feasibility of the study setting, the content validity of the tools in determining the clarity of questions, the effectiveness of the instructions, the completeness of response sets, the time required to complete the questionnaire, and the success of the data collection technique. The results obtained were useful in appraisal and modification of the tools; these subjects were not from the study sample.

Data verification was done using double-entry and proofreading methods.

We used Microsoft Office 365. Data were coded and then analyzed using the Statistical Package for Social Sciences (SPSS, version 20), and frequency, percentage, arithmetic mean for describing the central tendency of observation for each variable studied, and standard deviation for the measure of dispersion of results around the mean were used to present the data.

## 3. Results

Of the 306 HPs approached, 234 returned completed questionnaires, representing a response rate of 76.5%.  Table 1 demonstrates the sociodemographic characteristics of the participants. All the participants were full-time employees. Regarding the gender of the participants, 75.2% were female doctors. The mean age of the respondents was 29.1 years, and 53.8% of them were 25–34 years old. Of the total respondents, 34.6% had professional experience of more than 10 years.

In relation to methods used in normal labor, Table 2 shows that 44.9% of the respondents used nonpharmacological methods in the first stage of labor, whereas 18.4% used pharmacological obstetric analgesia methods in the first stage. The majority (67.9%) used pharmacological methods (e.g., oral or intravenous (IV) paracetamol or tramadol) or intramuscular opioids during the second stage of labor. The most frequent nonpharmacological method (such as family support, directed breathing and relaxation techniques, and massage) they used was to give assurance or explain the labor process (19.2%), followed by massage and therapeutic touch (10.3%) and changing maternal position or moving the mother around (8.5%); 70.1% of the respondents used pharmacological methods for pain relief (tablets and injections), whereas 12.4% were found to use epidural anesthesia and only 3.8% used nitrous oxide or inhaled gases. Fewer than one-quarter (19.2%) had taken a continuing education course that focused on pain-relief methods.

Table 3 shows the attitude of HPs toward the use of pain-relief methods during labor; the majority (89.7%) of respondents expected women to feel pain in labor and some expected that pain should be relieved (78.2%). The main reasons mentioned by 9% of the respondents (*n* = 21) who did not agree to pain relief were the following: (1) women did not ask for pain relief; (2) labor is a natural process; and (3) it could affect the baby, mother, or labor process. Of the participants, 42.3% believed that use of pharmacological methods might influence the progress of labor, and 84.6% thought that it increased women's comfort.

Of the HPs, 69.2% agreed that all methods will increase the ability of mothers to cope with pain.  Table 4 shows the different barriers to using labor pain-relief methods from the HPs' perspectives in Minia maternity units, using the four-point Likert scale. Most of the respondents thought that hospital-related factors are the main barrier against use of both pharmacological and nonpharmacological methods of pain relief. Other barriers included attitudes or beliefs followed by patient-related factors.

## 4. Discussion

To the best of our knowledge, this is the first survey in Egypt that we are aware of that explores HPs' perceptions and practices of pain relief for women in labor in the maternity units of Minia Governorate, highlighting that there is a conflict between the positive attitude toward pain relief during labor and the negative attitude toward use of pain-relief methods.

The survey also included questions to differentiate attitudes toward pain relief during the first and second stages of labor.

According to the American Society of Anesthesiologists (ASA) and American College of Obstetricians and Gynecologists (ACOG), the maternal request represents sufficient justification for pain relief [10, 11]. The ACOG also states that “labor results in severe pain for many women.”

A limited number of studies have been conducted in low-resource settings (LRSs) to assess HPs' options of pain relief in labor. In a study by Nwasor et al., a positive attitude toward the use of pain-relief agents during labor, coupled with the high awareness of the agents used, was found to be in conflict with the practice of providers. Fewer than half of the respondents (48.4%) provided any form of pain relief in labor even though almost 95% of them had attended a patient in labor in the 3 months preceding the survey [12]. In our study, the majority (89.7%) of HPs understood that women should expect pain during labor, and most of them (78.2%) believed that pain relief in labor is necessary. A result similar to our findings came from an Indian study in which the majority of respondents (92%) agreed that pain relief is required during labor [13]; 45% of obstetricians agreed with providing opioid analgesia for women in labor and considered opioids safe, noninvasive, easy to administer, and not requiring monitoring or the presence of an anesthetist [13]. However, in their conclusion, they stated that there is need for further awareness among HPs and liaison between women and both obstetricians and anesthetists to use effective pain-relief methods during labor.

Previous research has found some concerns about the use of pain-relief methods during labor. For example, a study done in Nigeria showed that women were worried about side effects on their babies (20%) or on themselves (17%), whereas some concerns were related to extra fees (3%) [14]. In our study, although 91% of HPs agreed to give pain relief during labor if facilities were available, 80.8% of them had some concerns regarding its use. Some reasons were attributed to availability, safety (for the mother and baby), the labor process, and community awareness. Previous studies have found these same concerns. On the contrary, 83.4% of women in a study conducted in South Africa showed little confidence in the method used in their settings, and 78% of the mothers had no concerns at all about the methods used [15].

In our study, the use of pharmacological methods for pain relief was 18.4% in the first stage and 67.9% in the second stage. This finding is in line with a study by Bitew et al. in 2016 that found the overall use of obstetric analgesia for labor pain management in Amhara Regional (Ethiopia) state referral hospitals was 40.1% [16, 17]. This proportion showed only the use of nonpharmacological obstetric analgesia methods. Even though the pharmacological usage was zero in this study, it is a common practice in many countries of the world.

The current study reported that epidural analgesia usage in labor was only 12.4%. Although epidural analgesia is the most common and complete method of pain relief available, most women surveyed in the 2006 Listening to Women Survey expressed interest in less-invasive methods. Recent reports support wider access to safe, less-invasive options for comfort and labor pain as part of a program to achieve improved maternal-child outcomes [16–19].

Gerdin, in his study that was conducted in Sweden between 1983 and 1986, reported that the use of the lumbar epidural was 16%, paracervical block was 12%, pethidine or morphine was 49%, and pudenda block was 62% [20].

In the present study, the most frequent nonpharmacological method that HPs used was giving assurance or explaining the labor process, massage, and therapeutic touch; breathing exercises were offered in the second part of nonpharmacological methods, followed by maternal positioning. The study by Madden found that the least preferred method by obstetricians was hypnosis and the least preferred method by midwives was the epidural. He also found that obstetricians had a greater preference for all pharmacological methods [21].

Analgesia for labor is widely used in high-income countries, but this is not the case in Africa [22]. Issues in high-income countries are focused on the choice of methods and complications. Its use is also affected by many factors, mainly including the relationship between the women and their HPs and the women's involvement [9]. In LRSs, the issue revolves around awareness, acceptability, and availability of analgesia for labor [6]. The priority in such settings is to provide evidence-based practices and care (including skilled birth attendance) rather than pain relief, which should be included in the delivery package as a promotion to women to access the health care facilities and to improve their quality of care.

The inclusion of pain-relief methods improves the quality of care, and the authors recommend working in a multidisciplinary fashion between different HPs to implement a suitable guideline that guarantees the safety, accessibility, efficacy, and acceptability of the chosen pain-relief methods. Raising awareness in the community and among HPs would also be helpful.

There were some limitations of our study; the study did not explore the women's views about pain relief in labor (health workers were asked but not patients) and did not include questions about the attitude toward using pain-relief methods in the postnatal stage or for women undergoing different modes of delivery, such as operative vaginal (forceps or vacuum) or abdominal (cesarean section) delivery. Also, we did not perform formal verification (validation) for this questionnaire, but it was tested by 5 experts in the field of obstetrics and gynecology and they agreed it. In addition, we did not set out any association between the HPs' gender, age, cadre, or place of work and the attitude toward pain relief. The small sample size is another weak point.

It is very clear that there is a wide gap between the use of pain-relief methods and women's need in Egypt. HPs understand the suffering of women during labor and agree to the use of pain-relief methods; however, the health care facilities are the main barrier. There is an urgent need for directed research to assess the attitude of women and HPs, especially in the LRSs, toward the use of pain relief during labor. The study of different barriers and proper assessment of the service provided, especially in the primary health care facilities, is mandatory. Raising the awareness of the community and HPs of the use of pain-relief methods as part of improving the quality of care during labor is also mandatory.

## Figures and Tables

**Table 1:** Sociodemographic characteristics of respondents.

Variable	Number	Percentage
*Hospital*		
Governmental	234	100.0

*Gender*		
Male	58	24.8
Female	176	75.2

*Age (years)*		
20–24	24	30.8
25–34	42	53.8
≥35	12	15.4

Mean age 27.17 ± 29.1 SD		

*Years of experience*		
Less than 5 years	25	32.1
From 5 to 10	26	33.3
More than 10 years	27	34.6

**Table 2:** Types of pain-relief methods.

Variable	Number	Percentage
*Methods used in the first stage*		
Nonpharmacological methods	105	44.9
Pharmacological methods	43	18.4
None	86	36.8
*Methods used in the second stage*		
Nonpharmacological methods	30	12.8
Pharmacological methods	159	67.9
None	45	19.2
*Types of nonpharmacological methods for pain-relief practice (total 135/234)*		
Heat or ice compression	13	5.6
Massage and therapeutic touch	24	10.3
Relaxing environment, audio analgesia (music, Quran, conversation, etc.)	15	6.4
Deep breathing/patterned breathing	18	7.7
Maternal positioning, waking, moving around the room	20	8.5
Giving assurance, explaining the labor process	45	19.2
*Types of pharmacological methods for pain relief used in labor (total 202/234)*		
Medications	164	70.1
Epidural anesthesia	29	12.4
Others/nitrous oxide or inhaled gases	9	3.8
*Continuous education that focused on methods for pain relief*		
No, I had not	63	80.8
Yes, I had	15	19.2

**Table 3:** Opinion and attitude of health professionals regarding the effect of pain-relief methods during normal labor.

Variable	Yes, *N* (%)	No, *N* (%)
Women should expect pain during labor.	210 (89.7)	24 (10.3)
Belief that pain relief in labor is necessary.	183 (78.2)	51 (21.8)
Use of pharmacological methods will influence the progress of labor.	99 (42.3)	135 (57.7)
Use of nonpharmacological methods for pain relief during normal labor is safer.	189 (77.8)	45 (19.2)
Use of pharmacological pain-relief methods will increase comfort of women.	198 (84.6)	36 (15.4)
Opinion regarding the effect of methods on the ability of women to cope with pain.	162 (69.2)	72 (30.8)
If resources are available and you have been asked to give pain relief, do you agree to give them?	213 (91)	21 (9)
Have you heard about the WHO analgesic ladder?	186 (79.5)	48 (20.5)
Do you have any concerns about using pain relief during labor?	189 (80.8)	45 (19.2)

WHO: World Health Organization.

**Table 4:** Barriers to use of labor pain-relief methods in health care settings, using the four-point Likert scale.

Barriers	*N* = 234			
	Disagree	Strongly agree	Agree	Strongly disagree
Patient-related factors	186 (79.5%)	21 (9%)	27 (11.5%)	0 (0%)
Clinician-related factors	54 (23.1%)	69 (29.5%)	75 (32.1%)	36 (15.4)
Hospital-related factors	0 (0%)	120 (51.3%)	111 (47.4%)	3 (1.3%)
Mixed	12 (5.1%)	153 (65.4%)	21 (9%)	48 (20.5%)

## Data Availability

The data used to support the findings of this study are available from the corresponding author upon request.

## Abbreviations

ASA: American Society of Anesthesiologists
ACOG: American College of Obstetricians and Gynecologists
HIC: High-Income Countries
HPs: Health Professionals
LRSs: Low-Resource Settings
TENS: Transcutaneous Electrical Nerve Stimulation.
